# Saudi women STEM pioneers: penetrating the mud ceiling

**DOI:** 10.3389/fpsyg.2024.1347944

**Published:** 2024-03-25

**Authors:** Amani K. H. Alghamdi, Hiya Almazroa

**Affiliations:** ^1^Department of Curriculum and Instruction, Faculty of Education, Imam Abdulrahman bin Faisal University, Dammam, Saudi Arabia; ^2^Department of Teaching and Learning, College of Education and Human Development, Princess Nourah bint Abdulrahman University, Riyadh, Saudi Arabia

**Keywords:** women in STEM careers, pioneers, Saudi Arabia, glass ceiling, mud ceiling

## Abstract

Although researchers actively study women’s experiences in STEM fields, few do so from women’s perspective. We thematically analyzed life narrative semi-structured interview data (46-item open-ended instrument, 90–120 Min) from eight STEM pioneering Saudi Arabian women careerists (mathematics, medicine, and biology) (convenience sample summer 2023). The objective was to glean their insights to discern self-reported influences (internal and external), struggles, and challenges in launching and advancing their careers. The extremely accomplished participants (all married, most with children) averaged age 65+, had 40+ years of experience and came from the three largest Saudi provinces. Important factors influencing choosing STEM included personality traits (e.g., deep desire to academically succeed; problem focused); secondary school peer/academic learning experiences; and male family member support, especially fathers. Struggles and challenges (often viewed as opportunities) included the mud (not glass) ceiling; male colleagues’ harsh, prejudiced treatment; and unsupportive administration. Participants were research driven and willing to relocate, re-educate, and change direction to establish and advance their careers. Implications for future research and policy initiatives are woven into the discussion and recommendations.

## Introduction

The issue of recruiting and retaining women in the Science, Technology, Engineering, and Mathematics (STEM) fields has been extensively studied and documented. During this scholarship, researchers addressed what has been termed *the leaky pipeline*, which encompasses the loss of a significant number of women across various pathways into and out of STEM university programs and careers (Glass et al., 2013; Liben and Coyle, 2014; Miller and Wai, 2015). The considerable body of literature on women’s engagement in STEM tends to focus on access and retention in secondary schools or tertiary education rather than the STEM workforce – career choice and attrition (Fassinger and Asay, 2006).

Also, the number of women employed in STEM has increased globally, but they remain underrepresented in most industries. The *Global Gender Gap Report* (WEF, 2022) ranked Saudi Arabia 127th out of 146 countries. Despite numerous studies on STEM *education* choices worldwide, limited studies have investigated the gender gap in STEM *careers* in the Gulf region (seven Arab states: Bahrain, Iraq, Kuwait, Oman, Qatar, Saudi Arabia, and the United Arab Emirate) (Patterson et al., 2021). The number of women in the Saudi STEM labor market remains low (WEF, 2016) even though most science, technology, and mathematics degrees are awarded to Saudi women, with engineering degrees mostly held by men (Al Gazali et al., 2013; Statista, 2023).

This gendered STEM inequality and underrepresentation also exists in the academy. The scientific community has documented a gap between the number of STEM research studies published by men versus women, The higher prevalence of men could be attributed to differences in productivity in STEM research. But Ross et al. (2022) discovered that a portion of this gap reflected the unacknowledged contributions of women as research team members. They were significantly less likely than men to be credited as authors on published research. Recent studies have thus concluded that women are not less productive, but rather their work is undervalued. To illustrate, Hofstra et al. (2020) analyzed new data on research teams and reported that women received less credit than men, with women systematically less likely to be named as authors on journal articles and patents.

In the Middle East, the pervasive pattern is high enrolment and graduation of women in STEM fields (40–70% depending on the nation) but markedly lower percentages for employment in STEM career paths (averaging 15%) (Islam, 2019). Also, Middle Eastern “women remain under-represented in the scientific research and those in the field receive less support and less promotion than their male counterpart” (Islam, 2019, p. 99). Moreover, they encounter the glass ceiling, which is an invisible systemic barrier that prevents women from rising to senior-level positions (Amon, 2017; Taparia and Lenka, 2022). That said, “women in the Middle East have been shattering numerous glass ceilings within contemporary inordinate length of time and traversed a frontier into the ‘formerly’ male-dominated world of STEM fields” (Islam, 2019, p. 103). This exciting time for Arab women inspired our Saudi study.

While efforts have been made to promote the advancement of women in STEM fields (e.g., education access, career paths, and research scientists), limited research directly examines this issue from a women’s perspective as they strategize and progress in male-dominated disciplines and career paths (Amon, 2017). And although a significant body of literature exists about female underrepresentation and undervaluation in STEM, most studies neglected to explore women’s lived experiences (to be discussed). To better understand the complexity of this phenomenon, our study aimed to highlight the diverse voices of accomplished Saudi women professionals in STEM with a combined field experience of well over 350 years. Our approach enabled us to capture meaningful experiences and viewpoints insufficiently documented in research (see next section).

## Challenges confronting STEM women

The global absence of women in STEM education and careers is well documented. Women tend to face barriers in their STEM career advancement in three different contexts: (a) socio-cultural barriers (e.g., stereotypes, social norms, sexual harassment); (b) education barriers (e.g., gender biased classrooms, and lack of female role models); and (c) work-stage barriers (e.g., blocked access to jobs despite similar qualifications, gendered pay gaps, biased performance evaluation and promotion, and family–work conflict and balance). These may differ in nature and impact depending on social and cultural contexts (Algaafreh and Sayilar, 2022).

In relation to STEM, gender, and role models (which is a powerful barrier), Cheryan et al. (2011) concluded that gender (male or female) of the role model may be less important than the extent to which role models embody current STEM stereotypes. The latter includes the assumption that STEM careerists are supposed to be male not female. Cheryan et al.’s (2011) results suggest that if male mentors reject this stereotype, they can successfully support and mentor aspiring female STEM students and professionals.

Regardless of the country, another significant barrier is the old boys’ club. Ruder et al. (2018) coined the phrase “patriarchal DNA” and noted that it permeated study and work environments making STEM women “often feel unwelcome, and unsatisfied with the rate of their accomplishments. [They] feel a sense of betrayal as their work is evaluated as being ‘less than’ the work of men. [And lateral violence and microaggressions can make them feel] subordinate to men” (p. 121). This men’s club either drives women out or drives STEM women to creatively adapt to a toxic environment so that they can succeed.

Many studies have examined, from various perspectives, the barriers and enablers women experience in STEM careers. However, limited studies have focused on *women’s* perspectives and strategies for advancing in male-dominated STEM fields. In one example, STEM women faced many barriers along their career path, one being the glass ceiling or the “glass obstacle course” (De Welde and Laursen, 2011, p. 576). In their study, barriers included (a) the “Old Boys’ Club” (i.e., the alleged advantage male employees have over female counterparts in interacting with powerful men); (b) a lack of female role models; (c) sexism and harassment (i.e., prejudice, discrimination, and stereotyping); and (d) difficulties in balancing work and personal life (De Welde and Laursen, 2011). These results align with Algaafreh and Sayilar’s (2022) work.

Prieto-Rodriguez et al. (2022) conducted interviews with Australian STEM women to understand what they faced, accepted, and valued on their individual career journeys. Although their journeys varied significantly, participants possessed similar personality traits and experienced common societal prejudices. Data revealed that resilience and determination were critical as was a strong, early interest in STEM. Women’s opinions were sharply divided on some issues, such as whether positive discrimination was a barrier or an enabler for their career (i.e., special measures for disadvantaged groups facing entrenched discrimination). Prieto-Rodriguez et al. concluded that, despite structural barriers, these women “survived” their work environment only through their determination, resilience, and fervent interest in STEM.

Stewart’s (2021) phenomenological research focused on conditions contributing to women’s underrepresentation in STEM leadership positions. Her study, using storytelling, involved 12 American women with unique journeys including secondary educators, postsecondary university professors, and professional STEM field careerists. Stewart wanted to reveal how self-efficacy develops during adolescence and influences persistence among women STEM leaders. A focus on adolescence was warranted because girls’ aspirations about their future education plans and occupational trajectories begin to solidify during early adolescence (Kim et al., 2018).

Stewart (2021) identified four common themes influencing self-efficacy: (a) shaping of mental exploration (i.e., wondering how the world works); (b) math efficacy (i.e., seeing the world through pattern, shapes, and numbers); (c) self-discovery through problem solving (using logic, inquiry, and questioning) about how things work; and (d) connecting to an encouraging learning community. Her study advances qualitative research about STEM women by allowing women to share their experiences in their own voices. She dedicated the study to adolescent “girls who may question their abilities, young women questioning whether to begin careers in STEM, and practicing STEM professionals seeking support in sustaining a career” (Stewart, 2021, p. 14).

Babalola et al. (2021) explored the organizational culture and challenges African women faced in STEM leadership positions. It is well known that the glass ceiling prevents STEM women from progressing as quickly as their male counterparts (De Welde and Laursen, 2011). Babalola et al. identified factors that inhibited African women’s pursuit of leadership in STEM fields. Their qualitative study in 12 African countries revealed that education level, scholarship, supportive organizational structures, commitment, hard work, and tenacity enabled STEM career path processes and attained positions. However, women experienced less acceptance than men in STEM leadership positions because the organizational culture in several African countries still devalues women as STEM leaders (Babalola et al., 2021).

A recent qualitative study of 30 Canadian women working in natural resource industries (e.g., energy, mining, and forestry) identified several barriers to recruitment, retention, and advancement in their STEM careers: (a) work commitment and competency inequitably being questioned after return from parental leave; (b) a lack of information and awareness of opportunities available in the sector (partly due to a lack of informal networks); and (c) an old boys’ club workplace culture where women experienced isolation, mockery, and sexual harassment (Baruah and Biskupski-Mujanovic, 2021).

To add to this American, Australian, African, and Canadian research, our Saudi study aimed to collect data from Saudi STEM women pioneers to (a) better understand the challenges they faced as STEM professionals, (b) gather insights into their educational and career trajectories and (c) explore contextual challenges and opportunities for women pursuing STEM studies and careers. Findings may empower Saudi women STEM professionals by enlightening them, so they can develop a different understanding of themselves as STEM careerists and professionals.

## Current STEM landscape for Saudi Arabian women

The underrepresentation of women in STEM fields is a global issue. Only 18% of women in higher education worldwide are studying STEM (Malek, 2021). Although that number is double in Saudi Arabia (38% of Saudi STEM graduates are women), only 17% work in the STEM sector. The most recent national development plan, *Vision 2030* (KSA, 2016), deeply colors the Saudi landscape on women in STEM. Although *Vision 2030* recognizes the need to increase education and training in STEM, it is relatively new to Saudi Arabia (Babineau, 2023). The Chief Information Officer (CIO) of a major Saudi university was recently quoted as saying “from what I see across the nation, there is definitely an emphasis on STEM. It is clearly recognized that these areas are the cornerstones for economic transformation” (Benito, 2023, para. 5).

As a strategy to achieve *Vision 2030’s* goals, the Saudi government intends to increase women’s workforce participation from 22 to 30% by 2030 (KSA, 2016), especially in STEM (Babineau, 2023). This strategy is working, as the overall labor force participation rate among Saudi females in 2022 had risen to nearly 30% (27.8%) (although still low compared to males, 80%) (World Bank, 2023). Achieving this goal will require continually addressing the barriers and challenges women face in the labor market including cultural norms, lack of access to education and training, and limited opportunities in certain fields. Encouraging and supporting women’s education and careers in STEM fields would play a significant role in achieving the government’s increased workforce participation goal and building a diverse and skilled workforce that can contribute to the country’s economic growth and development.

However, as Khaishgi (2019, para. 2) observed: “in the Kingdom, STEM-related jobs are limited at the moment, as the economy is primarily oil-based and there are few technical jobs available.” Moreover, although “almost 80 percent of (Saudi) girls were keen to study STEM… securing a job after the degree remains the challenge” (Khaishgi, 2019, para. 9). Also, a significant imbalance of Saudi women in STEM fields (careers) exists compared to the number of female STEM graduates (Islam, 2019).

To elaborate, despite nearly equal representation of Saudi men and women in STEM graduates, women’s participation in STEM in higher education is significantly lower than men’s (Malek, 2021). When considering individual STEM fields, the most popular fields for Saudi women are communications and information technology, physics, and biology while men enroll in engineering, architecture, and construction. Slightly more Saudi women were enrolled in mathematics and statistics than men (Khaishgi, 2019; Statista, 2023). This underrepresentation within higher education likely contributes to the lack of Saudi women in STEM fields in the current workforce. It is essential to address these disparities and increase women’s participation in STEM fields to allow for a more diverse and inclusive workforce, which can lead to better innovation and economic growth. Initiatives to promote and support women’s education and careers in STEM fields are necessary to achieve this goal.

In 2022, Saudi Arabia also reported the highest increase in gender parity in workforce participation among 14 countries, with a 0.097 increase (WEF, 2022). This is a positive sign that the nation’s efforts to promote gender equality and women’s participation in the workforce are having an impact. *Vision 2030* aims to reduce the country’s dependence on oil production. This marks a significant shift in the country’s focus on modernizing and reclassifying itself as a knowledge economy that requires highly skilled workers and more investment in education, especially STEM, to improve the employment rate. The government flagged education as a critical element in achieving this ambitious goal (KSA, 2016; Jawhar et al., 2022).

The current Saudi government has also prioritized reforming the status of women, who constitute nearly two thirds (58%) of Saudi university graduates. However, despite the high rate of female university graduates, the rate of females in the labor market with a bachelor’s degree is lower (62%) than men; that is, their labor rate participation is lower. By investing in education, particularly in STEM fields, and creating opportunities for women to enter and succeed in the STEM workforce, the country can eventually build a diverse and skilled workforce that can contribute to its economy’s growth and development. Encouraging and supporting women’s education and careers in STEM fields can also help address gender disparities and promote gender equality in the workplace, which have been identified as barriers to female STEM leaders (Algaafreh and Sayilar, 2022). Thus, gaining insights from women living the STEM education and career experience is a timely research initiative.

### Research question

*What insights can be gained about pioneering Saudi women engaged in STEM careers from collecting and analyzing their life and career narratives focused on important influences, struggles, challenges, and internal processes, especially reflection*?

## Method

### Sample frame

Criteria for inclusion in this qualitative study included (a) previously held or currently in a leadership position in a STEM profession, (b) a Doctoral degree in a STEM subject, (c) being recognized as a leader in the STEM field, (d) having studied and worked fulltime in a STEM field in Saudi Arabia and (e) being born before 1965. Potential participants were located through researchers’ personal connections (i.e., convenience and purposive sampling) and others’ recommendations. The intent was to sample participants who could best provide information related to the research question (McGregor, 2018).

The final sample frame comprised eight Saudi STEM career women. Adhikari (2021) recommended one to 25 participants for narrative studies with 12 being an average sample frame. Participants were from the three largest Saudi provinces: the central area Riyadh capital (conservative), the Eastern province (more conservative), and the western province Hijaz (most liberal) [characterized by Wagemaker (2012) and Alqefari (2015)]. They were aged 55–68 years (average 65+) and had worked in the STEM field since the 1980s. This represents 350 combined years of experience averaging 40+ years. All were married (three to non-Saudi men), and all but one had children (averaging three each). All held post-graduate qualifications in a STEM subject (i.e., medical, biology, or mathematics). Six attended schools abroad. One moved to SA for higher education. Six women (75%) had STEM patents (see Table 1 where the *W* attached to an alphabet letter for each participant stands for *STEM woman* and constitutes a protective pseudonym).

**Table 1:** Sample frame profile of Saudi STEM women (*N* = 8).

Pseudonym	Qualifications	Employment	Years in the field	Married # children	Age at time of study	Province
FW	Medical/PhD in radiology, patents	Retired from teaching –but practicing	45	Yes non-Saudi 1 child	65+	Eastern
MW	Medical/PhD, PharmD, MSc toxicology	Forensic sciences director of health-related center	40 plus	Yes Saudi no children	59	Eastern
EW	Science biology microbiology endocrinology, patents	Dean of a research institute	40 plus	Yes Saudi 5 children	59+	Eastern
BW	Medical/PhD in family medicine	Retired from teaching – but practicing	45	Yes Saudi 6 children	65+	Eastern
LW	Medical/ENT, Patents	Retired from teaching – but practicing	45	Yes non-Saudi 5 children	65+	Western
TW	Mathematician, patent 1st globally	Retired from teaching – but practicing	45	Yes Saudi 5 children	65+	Riyadh
HW	Biologist, patents	Retired from teaching– but practicing	40 plus	Yes non-Saudi 1 child	65+	Western
SW	Medical/ ophthalmologist, patents	Retired from government but teaching – practicing	50	Yes Saudi 3 children	65+	Riyadh

### Data collection protocol

The research method has been successfully employed in international studies on women’s careers (Babalola et al., 2021; Stewart, 2021). We strove to understand how Saudi women in STEM careers learned and developed from experiences that changed them: important influences, struggles, challenges, and internal processes, especially reflection. The research design involved thematically analyzing reflective life and career narratives collected through the lens of STEM women. The Institutional Review Board (IRB) at each authors’ affiliated university approved the study.

#### Interview instrument

We adapted Stewart’s (2021) interview instrument. The initial structured questions sought information about demographic data and work roles. The remaining 46 questions (see Table 2) were open-ended and designed to stimulate narratives around their lives and careers. The questions served as probes and were intentionally broad to allow participants freedom and flexibility in answering from their own perspective. Questions were designed to solicit information about their experiences related to their STEM career. They were encouraged to talk about factors that had influenced their career preferences from a young age through school and college to the present day.

**Table 2:** Questions for semi-structured interviews.

Family context	What were your parents’ levels of education and their occupations? What were some messages you received from your family regarding your work ethic, aspirations, and/or education? Were there any family traditions that impacted you during your adolescence? What type of responsibilities (e.g., chores) were required of you as an adolescent in and outside the home? What types of activities and/or programs did you participate in (e.g., sports, school clubs, and/or after-school programs)? How did you enjoy them? What did you enjoy about them? How did the activities make you feel?
Adolescent self-discovery	How did you perceive yourself as an adolescent girl? How were you perceived as an adolescent girl? By others? In school? At home? In the community? Please explain your journey of self-discovery as an adolescent girl. What specific experiences do you attribute to your self-discovery?
STEM interests and connection	Please reflect on your initial connection to science and explain that experience? How did the connection and experience make you feel? What about science prompted your initial interest as an adolescent? When and how did you realize you enjoyed science? Explain what triggered your curiosity about science. What was your initial takeaway from the experience? How did it make you feel?
Middle school, high school, and academic performance	What positive academic influences inspired you as an adolescent girl? How did they influence your performance? Why? Please share an experience you had as an adolescent in school when your academic achievement in the classroom was praised. How did it make you feel? What environmental influences (teachers, peers, family) contradicted your interest in science? What environmental influences (teachers, peers, family) supported your interest in science? Do you have any immediate family members and/or close relatives in science fields? If so, how did it impact or influence your interest in science? What environmental influences (teachers, peers, family) supported your interest in science? How did you perceive your academic ability in science compared to that of other students?
Adolescent experiences / journey	What were some of the adolescent experiences that helped shape your academic journey? What experiences specifically shaped your effort to persist academically in and outside the classroom? Please explain a situation during adolescence in which you experienced self-doubt that caused you to question your abilities and impacted your academic performance? What contributed to your self-doubt?
Adolescent challenges and influences	What challenges did you experience as an adolescent? In the classroom? Outside the classroom? What contributed to these challenges? What environmental factors influenced your academic journey? How did negative academic influences impact you as an adolescent girl in and outside the classroom?
Adolescent career aspirations	What career fields inspired you as an adolescent? As an adolescent, what aided and/or nurtured your interest to pursue a career in science?
Reflection on academic experience	During your academic journey, did you persist by choice or circumstance? Please explain your definition of academic persistence. What or who influenced your academic persistence? How? What were some of the experiences during adolescence that helped shape your academic persistence? At what point during your academic journey did you achieve self-mastery? What experiences do you attribute to your self-mastery? If you had the opportunity to speak to an adolescent girl with self-doubt about her ability to persist in science, what would you say?

#### Data collection

Data were collected from July to September 2023. Two participants were interviewed in person in a private setting, and six were interviewed via Zoom. Each formal 90–120-min interview was audio recorded with the participant’s permission (informed consent). During the interview, the interviewer (both authors conducted interviews) repeated back to the participant or summarized the recorded information to reaffirm the data’s completeness and accuracy (Stewart, 2021). Trained assistants transcribed the Arabic recordings after which the researchers performed member checking to establish both accuracy and credibility. Parts of the transcripts and quotes were translated to English for reporting in English-language publications. Pseudonyms assured confidentiality (Lincoln and Guba, 1986).

### Data analysis

The interview data underwent a thematic analysis. We *iteratively* read the transcripts following Owen’s (1984) coding protocol; that is, we identified and labelled initial themes (repeated patterns), searched for themes that may have been missed on previous readings, revised theme labels and descriptions, and re-categorized text segments. This protocol was repeated until we agreed on the common threads related to the research question (Owen, 1984 Braun and Clarke, 2006). We achieved high inter-rater reliability (agreement on coding) (0.89 coefficient) (Krippendorf, 1980). Direct quotes provide manifest evidence of a theme. Latent evidence is shared in the form of our interpretation of and inferences from the data. All is embedded within an analytic narrative which goes beyond describing the data and contains an argument in relation to the research question (Hancock, 2002 Braun and Clarke, 2006 McGregor, 2018).

## Findings

As in other studies (Madsen, 2010; Adhikari, 2021; Stewart, 2021), the life narrative approach was helpful in collecting data to answer the research question. This paper reports five of seven themes identified in our analysis: themes 1–2 relate to factors influencing STEM career choices and 5–7 focus on struggles and challenges in STEM career paths and leadership roles. For clarification, struggles deplete motivation and target innate abilities like patience and strength, while challenges trigger reflection and test and nurture potential (Madsen, 2010). Findings are presented first followed by our analysis, interpretation, and discussion of key points (McGregor, 2018).

### Theme 1: Role of familial responsibilities and men’s support in Saudi women’s STEM development and advancement

The thematic analysis revealed that many participants carried into their STEM careers childhood lessons learned from family responsibilities. These experiences would have occurred 40+ years ago in the early eighties. FW recalled that *“my mother taught me not to buy anything that I do not need. … I had a department head (DH) who made my experiences very difficult. [But I eventually became] the first female DH in a medical department in a Saudi teaching hospital. The skill my mother taught me helped me greatly to run a cost-effective post graduate program. I was also able to bring in a non-invasive treatment because I made it effective on an economic level…. I learnt from my mother.”* Conversely, HW admitted that “*I used to hide under the bed with a flashlight and finish my studies to escape household responsibilities [assigned by my mother]*.”

TW (a mathematician) recalled that “*my mother used to assign me the task of taking care of my younger sisters, and I used to help my mother with the housework in addition to studying. … I have known how to read and write since I was four years old.”* SW was the firstborn sister and *“given responsibility for my siblings from an early age. I also raised three children on my own while gaining [my medical degree in ophthalmology].”* Although not commenting on family responsibilities for her siblings, LW studied in a STEM field while creating and caring for her own family*. “I married in my third year of medical studies… and gave birth to my first child while studying. … I went to back to school three days after giving birth. … I used to leave my baby with my mother, but I was tired. I used to put pads on my breast for milk and change my blouse.”* She raised her children while embarking on and solidifying her STEM career in medicine, which includes registering a patent (Table 3).

**Table 3:** Prevalent themes identified in Saudi STEM women’s life and career narrative data.

Role of familial responsibilities and men’s support in Saudi women’s STEM development and advancement Adolescent-age factors influencing Saudi women’s STEM career choices Use of determination, resilience, and visionary mindset to navigate challenging STEM environments Mud ceiling instead of glass ceiling Inherently research driven and compelled to leave an impact

We found strong evidence of the positive influence of family support (notably men’s support – father and husband) on participants’ STEM career choices and success. Despite Saudi Arabia being a patriarchal and segregated society. LW remembered that “*my husband* [non-Saudi] *was very supportive during my college years.*” EW like-mindedly agreed that “*if not for God and my father [a lawyer who loved science], I would not have completed my master’s and doctorate. I had a scholarship for my master’s degree, but my father paid for my doctorate … and bought me very expensive books*.” BW valued “*the close presence of my father. Even though he was far away, he was always communicating with me by phone and would come to Riyadh to visit me rather than me going home and wasting my time travelling*.” At university, BW recognized the college’s male dean as “*a father figure … who inspired the small group of 13 female medical students.*”

TW’s father (“*an encyclopedia scholar who loves and appreciates knowledge*”) fostered “*my love of knowledge from the age of four*.” He contradicted and temporarily estranged his own mother (TW’s grandmother) when the latter asserted that *“girls do not study beyond grade six.”* He replied, *“of course they should study”* and asked TW’s older sister to take her to primary school and ask the principal to “*let her learn how to read and write*.” TW’s father also helped her brothers and sisters to “*create weekly magazines*.” She went on to become “*a writer for a Saudi newspaper for eight years and then the first woman at the university scientific council*.” Later in life, TW told her ill father she had been nominated for a prestigious award. “*He stood up and came before my head and kissed me and said to my mother, ‘Listen, … your daughter is making history.’ I mean the joy that made him happy. It was something unforgettable [even] more important than the nomination*.”

All HW’s siblings were girls, and “*my father was proud of us.* …*My father suggested that I go into biology. … He believed in me and the importance of my education and sent me abroad and approved of my stay overseas for higher education*.” FW similarly commented that both her father and husband strongly supported her on her STEM trajectory. “*My father approved my education in medical school although the extended family refused to mix with him*. … *My husband* [non-Saudi] *walked in carrying my doctoral thesis in his hands reflecting to my family his pride in my accomplishment*.” MW also said, “*my father was very keen on education. He was a judge and supported me to go to medical school. …But my departure from home had some kind of pressure on the family … and this is always the case*.”

### Theme 2: Adolescent-age factors influencing Saudi women’s STEM career choices

This theme focuses on factors that influenced participants’ career choices in STEM fields, especially while they were in secondary school. Three factors strongly influenced adolescent-age STEM career choices: curiosity, being easily bored, and an insatiable desire to academically exceed. These internal and external factors were often intertwined. They concerned (a) the lack of extracurricular activities and practical experiences in participants’ educational environments and (b) how this limited environment influenced their focus on academic pursuits. Also, (c) both limited resources and limited activities during their leisure time (away from the educational environment) influenced their intellectual engagement and academic pursuits, which even then inclined toward STEM.

FW (medicine and radiology) recalled that *“since my childhood I was curious, and I put my nose into everything.”* TW reminisced, “*there was nothing to do on vacation. I had fun with schoolbooks and solving math problems*. … *I went higher than my classmates jumping ahead because of my level*.” HW (biologist) explained that “*I had nothing to do but read. We saw nothing but fields and cows, and we had nothing to do. There was nothing else to do. I was studying all the time*. … *I went from 1 to 2 and from 4 to 6. I jumped grades in the system*.” BW recalled “*I went to summer school to take courses [I knew I would have to] take upon returning to school just so I was ahead in all subjects*.” She went on to specialize in family medicine.

From a different perspective, LW (medical STEM career with patents) “*was in the best and first female school in Jeddah. It had a high reputation and was distinguished in competitions and events. I always came first*.” This success inspired her. Her advice to today’s female STEM students, so they can always come first, is to “*choose a field you love [and] are passionate about no matter how many challenges you face.”* SW (medical eye doctor) noted how “*my male high school teacher gave us Algebra problems, and a mistake happened, and I raised my hand and corrected him. The principal called me into his office and said, ‘SW, we cannot leave you in the classroom.’ I thought they had expelled me. I was perplexed and said, ‘Why, what did I do?’ He said, ‘No, no, your intellectual level is higher than the other students,’ so they moved me to, what they called, independent studies* [*so my intellect could flourish*].”

EW recounted that while growing up she had *“loved medicine [but] did not look at sick children with great affection.”* She thus shifted gears “*from medical school to biology and physiology… to better serve humanity.”* Following her passion influenced her choice to pursue and succeed in a STEM career. LW commented on the social norm of wearing a niqab. She said, *“I proved myself veiled and* [*eventually learned*] *who respects you. They respect you because of your mind, because of your morals, because of your knowledge, and not because you look good* [i.e.*, wear ‘proper’ attire*].*”*

### Theme 3: Use of determination, resilience, and visionary mindset to navigate challenging STEM environments

This theme concerned (a) the participants’ ability to navigate challenging environments and their natural inclination toward leadership and taking initiative; and (b) situations that showcased their determination, resilience, and visionary mindset. To set up this theme, consider MW’s insightful comment: “*Any situation becomes a problem when you put it in the category of a problem, but when a topic is placed in the category of a challenge … an opportunity or opportunity can be benefited from in one way or another. The concepts are different.”* To reiterate, challenges trigger reflection and nurture and test one’s potential (Madsen, 2010).

In an early-life example of using perseverance and initiative to navigate a challenging STEM-related environment, TW encountered financial constraints when she was ready to enroll in high school. Her family lacked tuition money. “*What did I do? I sold the gold bracelets my father gave me when I graduated from primary school and used the money to pay the tuition of 200 riyals*.” She said that only six out of 20 students passed the secondary mathematics exam at the end of high school, “*me, my sister, and four others. Thank God [I sold the bracelets] and attended secondary stage*.” She added: “*I loved mathematics, had no problem with it, and was passionate about it*.” Her determination and resilience at a young age paid off. She is a career mathematician, with a global patent, retired from teaching after 45 years but is still active.

SW found a way to get around herself when she was younger, which led to a STEM career. She recalled: “*I wanted to be an ambassador to represent my country. My father told me instead that ‘it’s through medicine that you can represent your nation…not by being a women ambassador.’… I mean I could have worked as a consultant with an attractive salary, but instead I did a fellowship in pathology (in United States) for a year looking at cells…When I came back to Saudi Arabia, I spent three years completing a Saudi fellowship, an American fellowship, and a British fellowship.*” In effect, she set aside one dream and changed directions to embark on a STEM career path. She became a medical doctor in ophthalmology and went on to pioneer and establish an optical screening center that uses robotics.

Although she ranked in the top 10 in Saudi Arabia when she graduated high school and won a scholarship to study medicine in Pakistan, HW (biologist with patents) did not take advantage of it because *“I am afraid of blood. … My uncle, who had a business in England, said, ‘Do not worry. I will take care of you.’ When I came back to Saudi Arabia, a man who had proposed to me asked me to stop working. You see, in the seventies, men did not want their wives to work especially as I was in contact with men all the time in my work. [I eventually] married [but the male] university director had to agree to the marriage. Is this considered part of the challenges to STEM work or not*?” At a young age, she recognized the opportunity provided by temporarily moving to England and returning to Saudi Arabia to follow a biology STEM career path.

Even experiences with fellow adolescent students can be challenges to overcome when shaping a STEM career. SW, who was pulled out of regular classes because her “*intellect was higher than the other students*,” recounted that “*I did not even interact with my classmates during my teenage years. … It is possible that I was an outcast… because I was too smart. … When I was in middle school in America, they used to put me in the library and give me books. And I swear I remember they used to give me university-level algebra, and I would sit and write and settle [be content]*.” When she was at boarding school, HW said she was the youngest student (age 12 compared to 17 years). “*I sat in the front and the others wanted me to cheat for them. I do not like cheating. They would get annoyed with me*.” Despite this dynamic and her young age, HW persevered, graduated high school in the top 10 in the Kingdom and went on to become a pioneering Saudi woman STEM biologist.

LW recalled one instance when she and her fellow university female biology students “*held a protest and refused to take the exam and asked that we be allowed to study with the same professor who taught the male students [because the women had not been taught] very essential medical material*.” She further recounted that “*the first time I was a department head and a professor, the [male] professor who was there for the first two weeks … treated me like a monster. I would return to the hotel crying, but I was going for my goals, so I did not quit*.” In reference to another work environment, LW recounted that “*the male program director… treated me so tough… and it was very difficult, but I believe that difficult things make people more resilient*.” She endured this challenging treatment and persevered in her STEM career as a medical doctor specializing in cochlear implants (with patents).

### Theme 4: Mud ceiling instead of glass ceiling

This theme represents the constraints that societal norms and expectations impose on STEM women symbolized, and inspired by one participant’s metaphor, by the concept of a *mud ceiling* rather than a glass ceiling. Owen (1984) advised that one poignant quote can serve as evidence of a theme. SW’s powerful words exemplify the mud ceiling theme. She said, “*when our relatives drove us (we were four girls and one boy) from the airport coming from the US back to SA (Riyadh), they said, ‘If these girls were boys, what would they do for the country?’ Back then I promised myself that I would be like a Saudi man and do even better.* … *Women are always fighting barriers. In the West, it’s the glass ceiling. If they break it, it can be hurtful. Our ceiling is made of mud; we can only penetrate through the mud…and we did!”*

SW continued, “*every day is a challenge; do not stop. I will stop my life if I stop myself. I would like to take on every challenge…We do not have a glass ceiling in Saudi Arabia – we have a clay ceiling. Fail and you can still succeed and break through the mud [instead of breaking glass].”* LW passionately declared “*By God, I have always told them that I considered myself to be digging in the rock, but God has not made [women] stop, [so I think God intended women to have power]. … I [persevere] for the [sake of female] doctors who are with me. I tell them ‘Always do what you want. Do not feel that you are no one because you are a woman … Prove yourself.’*”

Societal norms and their impact on Saudi STEM women facing the glass ceiling were evident in FW’s comments as well. “*Another incident from the seventies that I cannot forget was when some man [was incredibly disrespectful] because I had my face uncovered as a medical student working in a mixed environment. Even my family did not know I was working with my face uncovered.*” She seems to have broken through this ceiling as evidenced in her comment about an experience 20 years later. “*I interned in emergency radiology during the 91 Gulf War. I was lucky to view the X rays of SS Iraqi young and old patients from the war… I saw all kinds of verities in illnesses in one year. … I was lucky enough to have many [such] opportunities that allowed me to move up faster [in my STEM career] than my male colleagues.”* In effect, FW breached the glass ceiling.

EW (biologist) recounted another form of glass ceiling – one where male leaders initially blocked what *she* envisioned as constituting science. “*During my first scholarship in the College of Science, I wanted to build animal cages and greenhouses. [The male leaders] said ‘that is something that concerns you, and do not bother us.’ I was told … that ‘if we need empty land to [build these structures], there will be difficulties.’ They also said that ‘education comes first and that this is something special about science’ [meaning not worth building].”* EW clarified that “*the new structures [cages and greenhouses] were for scientific research purposes and not for self-motives. [When the male leaders said no], we women actually began to prepare them ourselves. … After that, the male support opened. … They did move the animal cages to the college roof because of the odor* [laughter]*.”*

Although she did not call it such, LW confronted the old boys’ club syndrome in one of her positions and found it very challenging. It initially stymied her ability to do her job. *“So, they get together and play cards and have dinner with each other and find out things with each other and joke with each other and say things that we can say but do not know how to say, and they get along with each other.”*

### Theme 5: Inherently research driven and compelled to have an impact

Participants concurred that scientific research is a fundamental aspect of STEM career progress. This theme reflects their conviction that research is essential and not merely optional or a personal preference. FW loved doing research, knew how important it was to stay current, and spent her own money to procure research papers, reports, and journal articles. *“I loved doing literature reviews… I had to pay a great deal of my salary back then (approximately $2000 USD) to request the most updated research articles in my field …I called upon libraries abroad to pay for research articles. … Even ARMCO [state-owned petroleum and natural gas company] was a great deal of hassle to get a research paper from because I was a STEM woman.”*

Similarly, MW reported: *“I have approximately 25–26 research papers published in big international journals. … I know exactly that every paper I published was the result of a problem that I helped solve using research. [Because of this research], I belong to many organizations and am sitting with the most prominent Saudi woman specialized in my field.”* Her research is making a difference – having an impact.

EW believed that *“if you are at university, you must do research, even if it is not your passion. Scientific research is essential and not a preference. Research first, then education. Scientific research is a priority …. But there were clashes because there was no system to encourage women researchers,”* which meant that although research was important and had to make an impact, some Saudi STEM women were thwarted in their efforts to contribute. This aspect of the theme reflects challenges faced due to inadequate administrative and systemic support that encourages and enables STEM women to engage in research initiatives.

TW was honored that her “*research was referenced by thousands of people… When I applied for promotions, … I used to make a list of research papers that had in their title [reference to my unique contribution to the field], and I would list Doctoral dissertations and master’s thesis that did the same.”* All university tenure and promotion systems require faculty to provide evidence that their research is having an impact. TW further explained that she has “*31 scientific papers. I wrote a book at the beginning of my sabbatical year. Before I retired. …It is very valuable because it is more research-based in the sense that it is the history of every mathematical problem… It is like a research encyclopedia of the field.”*

MW knew from an early age that STEM research was imperative. “*I focused on creating a scientific environment and finding a practical application for the things that students study. [This] was an obsession of mine. I mean, I did not have that at the beginning of my life, so I was hoping that I would provide it for those who come after me.”* Her STEM research now enables her to *“represent my country in the World Health Region, and I am the only one from the Middle East.”* She has persistently engaged in research that has made a difference.

## Discussion and recommendations

As a caveat, recommendations for future research are woven into the discussion. Important influences shaping Saudi women’s STEM trajectory included (a) personality traits (e.g., problem focused, easily bored, abiding interest in science or mathematics, and a deep desire to academically succeed); (b) secondary school peer/learning experiences, including the school’s ability to provide intellectual stimulation; (c) support from male family members especially fathers instead of mothers; and (d) academic male mentoring instead of female mentoring.

Algaafreh and Sayilar (2022) reported that barriers to women’s STEM careers can be found in three areas: socio-cultural, educational, and work, which were supported by our findings. In varying degrees, Saudi STEM women pioneers commented on social norms, biased classrooms and learning environments, and blocked access to and lack of administrative support for career advancement. They stoically acknowledged that Saudi women are always fighting barriers, so why should Saudi STEM women be any different?

### Mud ceiling

One of the most compelling findings was the neologism – *the mud ceiling*. The literature recognizes the proverbial glass ceiling as a STEM challenge requiring perseverance, resilience, and tenacity. This metaphor symbolizes being able to *see* the top but being blocked from getting there. It is an invisible systemic barrier that advantages men while preventing women from advancing in their careers (Amon, 2017; Taparia and Lenka, 2022). Although some of our participants directly referred to the glass ceiling, which De Welde and Laursen (2011) alternatively called “the glass obstacle course” (p. 576), one ingeniously coined the term *mud ceiling* (Themes 4 and 5).

“The glass obstacle course metaphor is more accurate than the ‘glass ceiling’ one, because, unlike a ceiling, female STEM professionals encounter informal and formal barriers at every turn in their careers — from grade school to post-secondary education, to field work, and tenure and grant applications” (Sorokina, 2020, para. 7). A mud ceiling metaphor could suggest being unable to *see* higher-up positions let alone break through sticky soil to get there. That said, some participants did acknowledge *the mud* (meaning they could *see* it) and knew they wanted/needed to get through it. They did not perceive it as impenetrable despite not being to see through. Indeed, they said it was easier to penetrate mud (force a way through) than break through glass. Mud and clay are dense yet malleable – they can be reshaped by hammering or exerting pressure (i.e., persistence, determination, and tenacity). Middle Eastern STEM women are shattering the glass ceiling (Islam, 2019), but what would penetrating mud ceilings look like from a Saudi STEM women’s perspective? This compelling finding warrants further research.

For example, while interpreting this finding, we discovered that some scholars outside KSA have used the mud metaphor to discuss STEM women, but they viewed mud as a floor and not a ceiling. In her discussion of why Silicon Valley treats STEM women so awful, Mundy (2017) quoted an informant as saying, “We’re going to have to go through this mud together.” When using a flamingo metaphor (they thrive in mud flats) while discussing women STEM leaders, Randell and Yerbury (2020) reflected on mud’s relatively hostile environment (unseen under the water) and said it was not “too much of a stretch to consider that [STEM] female leaders [must also] survive and thrive in [the mud] – what can be perceived as difficult circumstances” (p. 7). In his doctoral work about STEM women, Bing (2021, p. 161) referred to an ancient Chinese adage: “going through mud without getting stained.” This could refer to STEM women engaging with STEM men (i.e., going through the mud) without being thwarted, stymied, and defeated (i.e., the old boys’ club).

When used as metaphors, Gilkey (2023) described ceilings as “false limits that are imposed on us from outside, which we eventually accustom ourselves to, and which limit our ability to rise to the natural level we might belong at” (para. 5). In contrast, floors act as a stabilizing force, *but* what people consider safe and comfortable can sometimes surprise them and limit their growth and progress. Gilkey suggested that people’s ability to break through either floors or ceilings depends on whether they can make themselves seen and heard. He believed that moving through the mud (*per se*), or “leading through a turnaround” (2023, para. 13), can happen if women embrace challenges rather than balk and back down in fear.

It seems the fearless Saudi STEM women pioneers in our study were finding ways to navigate through the mud and clay, which some viewed as a ceiling but never a floor. It was not a stabilizing force (floor) that surprised and confounded them, but a false limit (ceiling) imposed on them by others. Self-framing their STEM challenges as a *mud ceiling* suggests they did not relish standing under falling and shattered glass (if they break through the glass ceiling), preferring instead to adhere to the Chinese adage of moving through a mud ceiling without getting stained. This theme and these discussion points are quite fascinating and warrant further conceptualization and theorization.

### Adolescent experiences

Adolescent experiences seemed to be very formative for our study participants (Theme 2). Our findings concurred with Kim et al.’s (2018) assertion that girls’ aspirations about their future STEM-related education plans and occupational trajectories begin to solidify during early adolescence. Our findings further affirmed Stewart’s (2021) concern for self-efficacy during adolescence (i.e., confidence in one’s ability to exert control over one’s motivation and behavior) and its influence on STEM women’s success. Like Stewart’s (2021) findings, some Saudi participants (a) valued self-discovery and learning through problem solving and (b) loved and demonstrated math efficacy (i.e., thinking in patterns and numbers), both of which increased their self-efficacy. Their adolescent confidence and self-efficacy carried into their STEM careers and stood them in good stead as they progressed. Saudi educators should heed this finding and develop strategies to bolster female adolescents’ self-efficacy while promoting STEM careers.

### Male support and mentoring

An unexpected finding was participants’ frequent mention of the profound importance of their father’s support and that of academic male mentors (Themes 1 and 2). Previous research repeatedly confirmed the importance of STEM career women having female role models and mentors if they aspire to thrive and advance in their STEM careers (De Welde and Laursen, 2011; Aljaafreh and Sayilar, 2022). However, our research provided a different perspective. Saudi women embarked on STEM careers because of men’s influence and undaunting support, not because of other women’s support. That said, several participants commented that they were committed to supporting other STEM women coming behind them or working beside them.

Our findings also support Cheryan et al.’s (2011) presumption that the gender of role models may be less important than the extent to which role models embody STEM stereotypes. Several women in our study attributed their success to male role models, which suggests the latter eschewed STEM stereotypes and successfully mentored aspiring female STEM students and careerists. Future non-Saudi studies about women STEM careerists are encouraged to explore the role of male mentors and role models within their context and then conduct cross-cultural and comparative analyses to tease out this finding.

### Liberal vs. conservative social norms

Regarding social and cultural norm barriers on STEM careers (Aljaafreh and Sayilar, 2022), participants who specifically mentioned their supportive husbands (Theme 1) were from a more liberal part of Saudi Arabia. Alqefari (2015) explained that people living in more cosmopolitan cities (such as Riyadh, Jeddah, and Dammam) have more exposure to Western culture. They are considered liberal because their thoughts are “more broadly informed, incorporating concepts derived from (international) politics and philosophy” (Wagemaker, 2012, p. 14). The more conservative regions are “religiously and culturally conservative” (Alqefari, 2015, p. 235) and have stricter enforcement of norms around gender segregation, gender attitudes, and gender learning than cosmopolitan regions (Alqefari, 2015). Future research should consider the liberal/conservative aspect of social norms shaping Saudi women STEM careerists, so that these insights can be used when mentoring young women into STEM career paths.

### Research commitment

Findings affirmed participants’ commitment to the research enterprise, with most providing a count of published articles (averaging 30+), books, and patents (Theme 5). Participants also encouraged aspiring female STEM colleagues to engage in research and to publish. It is imperative, not an option or a personal preference. That said, participants’ research success seems to contradict that reported in the literature, where STEM women had fewer publications because they were undervalued not because they were less productive (Hofstra et al., 2020; Ross et al., 2022). Our participants were very research productive.

However, heeding our participants’ advice to commit to research may remain problematic for future Saudi female STEM careerists, because Middle Eastern women persistently remain underrepresented in scientific research (Islam, 2019). Nonetheless, listening to the life narratives of these Saudi pioneer STEM women may be a powerful way forward for aspiring women STEM professionals. They can come to appreciate that a strong commitment to and pride in research as a career imperative will contribute to their STEM career development and success.

### Old boys’ club

Findings strongly support our inference that the Saudi women pioneers in our study succeeded because of their determination, resilience, tenacity, and sustained interest in STEM (Themes 4 and 5). Prieto-Rodriguez et al. (2022) reached a similar conclusion in their Australian study, as did Babalola et al. (2021) in their African study. Our participants shared various examples of the old boys’ club syndrome, which is a common challenge most STEM women face and demands staunch tenacity (De Welde and Laursen, 2011; Ruder et al., 2018; Baruah and Biskupski-Mujanovic, 2021).

Insidious lateral violence and microaggressions in the old boys’ club can press STEM women into an adaptive mode (Ruder et al., 2018), which requires resilience and other traits manifested in our study. Instead of hindering them, it seemed to embolden them. They found workarounds to their advantage. Saudi Arabia is traditionally a decidedly masculine culture (Hofstede, 2015), which means Saudi women (including female STEM careerists) are likely used to, and must continue to anticipate, navigating the old boys’ club. Fortunately, Middle Eastern STEM women are bravely crossing “into the ‘formerly’ male-dominated world of STEM fields” (Islam, 2019, p. 103). As one participant said, aspiring Saudi STEM women should not let being a woman stop them. Future researchers should continue to monitor the impact of a men’s-culture and old boys’ club mindset on Saudi STEM women.

### Study limitations and future research suggestions

Our study intentionally focused on pioneers, so future researchers should engage younger generations to gain a more contemporary profile of Saudi-women-in-STEM narratives. The sample size was understandably small because there are so few Saudi-women STEM pioneers. Future studies should expand their sample size to 30 or more women, a strategy that would correlate with studies cited in this paper where sample sizes ranged from 12 to 42. Qualitative research is incredibly valuable for discerning narratives, but future research should include a mixed methods design, so quantitative data are also available.

Women in our sample tended to be from more conservative regions of Saudi Arabia. Future studies should have a more balanced representation of conservative and liberal regions. Future researchers should also continue to employ a combination of purposive and snowball sampling. And despite the interview instrument asking participants to address obstacles and challenges, the tenor of our data set was positive. Future researchers may want to intentionally probe for instances when participants encountered obstacles and challenges that they could not overcome – a sort of counter narrative.

## Conclusion

This is the first study to provide insights into Saudi women pioneers’ lived experiences with STEM careers, which augments the longstanding focus on women’s underrepresentation and undervaluation in STEM (Amon, 2017; Babalola et al., 2021). And it addressed the lack of research in the Gulf region about women’s STEM careers (Patterson et al., 2021). Although STEM is relatively new to Saudi Arabia (Babineau, 2023; Benito, 2023), these eight pioneering women assuredly paved the way for future female STEM careerists, especially in medicine, mathematics, and biology, which are the most common STEM degrees granted to women in Saudi Arabia (Khaishgi, 2019; Statista, 2023).

For the last 40+ years, working into retirement and beyond, these pioneers have traversed their respective fields as trail blazers – they broke barriers at the frontiers of research; made astounding, ground-breaking discoveries, and achievements (patents); anticipated even welcomed challenge; and encouraged Saudi STEM women following similar paths to stay the course. Their main takeaway was, “*Do not feel you are no one because you are a woman. Choose a STEM field you love no matter how many challenges you face. Prove yourself.*”

## Data availability statement

The raw data supporting the conclusions of this article will be made available by the authors, without undue reservation.

## Ethics statement

The studies involving humans were approved by the IRB at Princess Nourah bint Abdulrahman University. The studies were conducted in accordance with the local legislation and institutional requirements. The participants provided their written informed consent to participate in this study. Written informed consent was obtained from the individual(s) for the publication of any potentially identifiable images or data included in this article.

## Author contributions

AA: Conceptualization, Data curation, Formal analysis, Methodology, Writing – original draft, Writing – review & editing. HA: Conceptualization, Data curation, Formal analysis, Investigation, Methodology, Writing – original draft, Writing – review & editing.
